# [*N*′-(3-Meth­oxy-2-oxidobenzyl­idene-κ*O* ^2^)benzohydrazidato-κ^2^ *N*′,*O*]tris­(pyridine-κ*N*)cobalt(III) perchlorate

**DOI:** 10.1107/S1600536811004715

**Published:** 2011-02-12

**Authors:** Gui-Miao Yu, Xiu-Yun Yang, Yuan Wang, Ya-Juan Xiao, Yun-Hui Li

**Affiliations:** a School of Chemistry and Environmental Engineering, Changchun University of Science and Technology, Changchun 130022, People’s Republic of China

## Abstract

In the mononuclear title compound, [Co^III^(C_15_H_12_N_2_O_3_)(C_5_H_5_N)_3_]ClO_4_, the Co^III^ ion is coordinated by three pyridine mol­ecules and one *N*′-(3-meth­oxy-2-oxidobenzyl­idene)benzohydrazidate Schiff base ligand in an *O*,*N*,*O*′-tridentate manner. The Co^III^ ion adopts a distorted CoN_4_O_2_ octa­hedral coordination environment.

## Related literature

For applications of Schiff base compounds, see: Ando *et al.* (2004 ▶); Guo *et al.* (2010 ▶). For the preparation of the Schiff base, see: Pouralimardan *et al.* (2007 ▶); Sacconi (1954 ▶). For related structures, see: Monfared *et al.* (2009 ▶); Sun *et al.* (2008 ▶); Yu, Zhao *et al.* (2010 ▶); Yu, Li *et al.* (2010 ▶); Zhang *et al.* (2004 ▶); Zou *et al.* (2010 ▶).
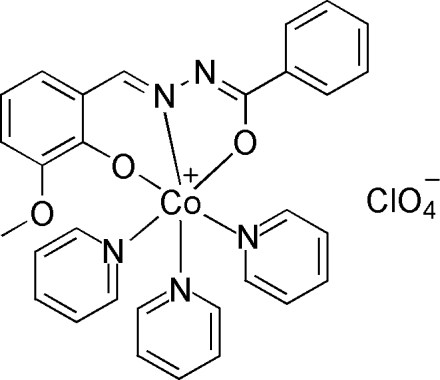


## Experimental

### 

#### Crystal data

[Co(C_15_H_12_N_2_O_3_)(C_5_H_5_N)_3_]ClO_4_
*M* *_r_* = 663.95
Monoclinic, 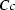

*a* = 10.7591 (5) Å
*b* = 13.2318 (6) Å
*c* = 21.0558 (10) Å
β = 94.610 (1)°
*V* = 2987.9 (2) Å^3^
*Z* = 4
Mo *K*α radiation
μ = 0.72 mm^−1^
*T* = 185 K
0.20 × 0.18 × 0.12 mm

#### Data collection

Bruker APEXII CCD area-detector diffractometer
Absorption correction: multi-scan (*SADABS*; Bruker, 2004 ▶) *T*_min_ = 0.869, *T*_max_ = 0.919
7543 measured reflections
4991 independent reflections
4431 reflections with *I* > 2σ(*I*)
*R*_int_ = 0.027

#### Refinement

*R*[*F* ^2^ > 2σ(*F* ^2^)] = 0.036
*wR*(*F* ^2^) = 0.072
*S* = 1.00
4991 reflections
398 parameters
2 restraints
H-atom parameters constrained
Δρ_max_ = 0.35 e Å^−3^
Δρ_min_ = −0.29 e Å^−3^
Absolute structure: Flack (1983 ▶), 2332 Friedel pairs
Flack parameter: 0.011 (12)

### 

Data collection: *APEX2* (Bruker, 2004 ▶); cell refinement: *SAINT* (Bruker, 2004 ▶); data reduction: *SAINT*; program(s) used to solve structure: *SHELXS97* (Sheldrick, 2008 ▶); program(s) used to refine structure: *SHELXL97* (Sheldrick, 2008 ▶); molecular graphics: *DIAMOND* (Brandenburg, 1999 ▶); software used to prepare material for publication: *SHELXL97* and *publCIF* (Westrip, 2010 ▶).

## Supplementary Material

Crystal structure: contains datablocks I, global. DOI: 10.1107/S1600536811004715/mw2001sup1.cif

Structure factors: contains datablocks I. DOI: 10.1107/S1600536811004715/mw2001Isup2.hkl

Additional supplementary materials:  crystallographic information; 3D view; checkCIF report

## Figures and Tables

**Table 1:** Selected bond lengths (Å)

Co1—O2	1.866 (2)
Co1—N2	1.864 (3)
Co1—O1	1.898 (2)
Co1—N5	1.957 (3)
Co1—N3	1.977 (3)
Co1—N4	1.987 (3)

**Fig. 1.**
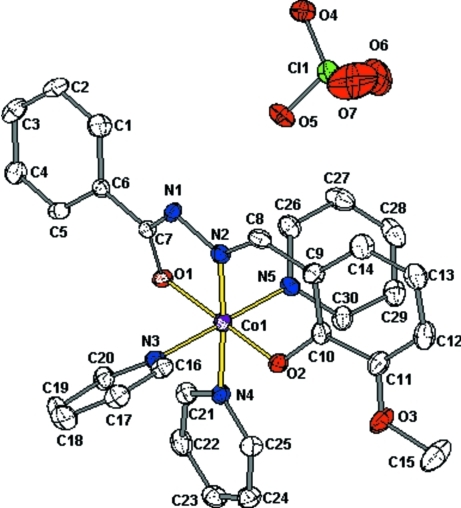
A view of the title compound. Displacement ellipsoids are drawn at the 40% probability level.

## supplementary crystallographic information

### Comment

The development of routes and strategies for the design and construction of Schiff base compounds are of great interest not only because of their intriguing structural motifs but also because of their important applications in antitumor activities (Ando *et al.,* 2004), magnetochemistry (Guo *et al.*, 2010), and so on. Acylhydrazone ligands are widely used to assemble coordination polymers, which have received a considerable interest over the last decade. From the structural point of view, selection of the Schiff base ligand 3-methoxysalicylaldehyde benzoylhydrazide(H_2_*L*) is a good choice for construction of coordination polymers with defined geometry and special properties, due to its containing a combination of nitrogen and oxygen donor atoms (Yu, Zhao *et al.*, 2010). Some geometrically intriguing supramolecular structures derived from this ligand have been reported including structurally characterized species with Mn_2_ (Yu, Li *et al.*, 2010), Cu_4_ (Monfared *et al.*, 2009), Fe_1_ (Zou *et al.*, 2010) units among others. As a continuation of our efforts on this system, we report the synthesis and characterization of the title cobalt(III) compound.

The molecular structure of [Co^III^(C_15_H_12_N_2_O_3_)(C_5_H_5_N)_3_]ClO_4_, together with the atom-numbering scheme, is illustrated in Fig. 1. Selected bond lengths are given in Table 1. The asymmetric unit of the title compound consists of a mononuclear cation [Co^III^(C_15_H_12_N_2_O_3_)(C_5_H_5_N)_3_]^+^, accompanied by one perchlorate anion. Several mononuclear compounds with similar structures have been reported previously (Sun *et al.*, 2008; Zhang *et al.*, 2004). The cobalt(III) atom has a distorted octahedral geometry, which consists of two oxygen atoms (O1 and O2) and one nitrogen atom (N2) of *L*^2-^ and three nitrogen atoms (N3, N4 and N5) from three pyridine molecules. In the ligand, the angles for the equatorial donor atoms [82.99 (11)° for O1—Co1—N2 and 93.38 (11)° for O2—Co1—N2] correspond, respectively, with the more constrained five-membered chelate ring O1—C7—N1—N2—Co1 and the less constrained six-membered ring N2—C8—C9—C10—O2—Co1. The N_2_O_2_ equatorial plane, defined by O1 O2, N3 and N5, shows a small but significant tetrahedral distortion. The maximum displacements from the least-squares plane through atoms O1, O2, N3 and N5 are 0.027 (3)and 0.025 (3) Å for atoms O1 and O2, respectively; Co1 is 0.0396 (4) Å below this plane.

### Experimental

The 3-methoxysalicylaldehyde benzoylhydrazide ligand (H_2_*L*) was prepared in a manner similar to the reported procedures (Pouralimardan *et al.*, 2007; Sacconi, 1954). The title compound was synthesized by adding Co(ClO_4_)_2_.6H_2_O (0.2 mmol) to a solution of H_2_*L* (0.20 mmol) in methanol 20 ml. The resulting mixture was stirred at room temperature to afford a dark brown solution. After 10 min pyridine (1 ml) was added and the solution was stirred for 3 h. Slow evaporation of the resulting dark brown solution over three weeks afforded brown crystals of the product.

### Refinement

All H atoms were placed in calculated positions and refined using a riding model [C–H (aromatic) = 0.95 Å; C–H (CH_3_) = 0.98 Å; and *U*ĩso(H) = 1.5*U*eq(C)]. The displacement ellipsoids for O6 and O7 are significantly larger than those of their neighbors suggesting some degree of disorder in this side of the anion however attempts to model this disorder with a split-atom model proved unsatisfactory.

### Crystal data

**Table:**

[Co(C_15_H_12_N_2_O_3_)(C_5_H_5_N)_3_]ClO_4_	*F*(000) = 1368
*M**_r_* = 663.95	*D*_x_ = 1.476 Mg m^−^^3^
Monoclinic, *C**c*	Mo *K*α radiation, λ = 0.71073 Å
Hall symbol: C -2yc	Cell parameters from 4029 reflections
*a* = 10.7591 (5) Å	θ = 4.9–50.1°
*b* = 13.2318 (6) Å	µ = 0.72 mm^−^^1^
*c* = 21.0558 (10) Å	*T* = 185 K
β = 94.610 (1)°	Block, brown
*V* = 2987.9 (2) Å^3^	0.20 × 0.18 × 0.12 mm
*Z* = 4	

### Data collection

**Table:**

Bruker APEXII CCD area-detector diffractometer	4991 independent reflections
Radiation source: fine-focus sealed tube	4431 reflections with *I* > 2σ(*I*)
graphite	*R*_int_ = 0.027
φ and ω scans	θ_max_ = 25.1°, θ_min_ = 1.9°
Absorption correction: multi-scan (*SADABS*; Bruker, 2004)	*h* = −12→12
*T*_min_ = 0.869, *T*_max_ = 0.919	*k* = −15→10
7543 measured reflections	*l* = −24→25

### Refinement

**Table:**

Refinement on *F*^2^	Secondary atom site location: difference Fourier map
Least-squares matrix: full	Hydrogen site location: inferred from neighbouring sites
*R*[*F*^2^ > 2σ(*F*^2^)] = 0.036	H-atom parameters constrained
*wR*(*F*^2^) = 0.072	*w* = 1/[σ^2^(*F*_o_^2^) + (0.0182*P*)^2^] where *P* = (*F*_o_^2^ + 2*F*_c_^2^)/3
*S* = 1.00	(Δ/σ)_max_ = 0.001
4991 reflections	Δρ_max_ = 0.35 e Å^−^^3^
398 parameters	Δρ_min_ = −0.29 e Å^−^^3^
2 restraints	Absolute structure: Flack (1983), 2332 Friedel pairs
Primary atom site location: structure-invariant direct methods	Flack parameter: 0.011 (12)

### Special details

**Table:**

Geometry. All esds (except the esd in the dihedral angle between two l.s. planes) are estimated using the full covariance matrix. The cell esds are taken into account individually in the estimation of esds in distances, angles and torsion angles; correlations between esds in cell parameters are only used when they are defined by crystal symmetry. An approximate (isotropic) treatment of cell esds is used for estimating esds involving l.s. planes.
Refinement. Refinement of *F*^2^ against ALL reflections. The weighted *R*-factor *wR* and goodness of fit *S* are based on *F*^2^, conventional *R*-factors *R* are based on *F*, with *F* set to zero for negative *F*^2^. The threshold expression of *F*^2^ > σ(*F*^2^) is used only for calculating *R*-factors(gt) *etc*. and is not relevant to the choice of reflections for refinement. *R*-factors based on *F*^2^ are statistically about twice as large as those based on *F*, and *R*- factors based on ALL data will be even larger.

### Fractional atomic coordinates and isotropic or equivalent isotropic displacement parameters (Å^2^)

**Table:**

	*x*	*y*	*z*	*U*_iso_*/*U*_eq_	
Cl1	0.16794 (8)	0.46289 (8)	0.39641 (4)	0.0359 (2)	
Co1	0.43297 (3)	0.52399 (3)	0.15848 (2)	0.02070 (11)	
C1	−0.0228 (3)	0.4207 (3)	0.15340 (18)	0.0328 (9)	
H1A	0.0004	0.3967	0.1952	0.039*	
C2	−0.1460 (3)	0.4165 (3)	0.12945 (19)	0.0374 (10)	
H2	−0.2070	0.3888	0.1546	0.045*	
C3	−0.1808 (3)	0.4523 (3)	0.06960 (19)	0.0366 (9)	
H3	−0.2658	0.4495	0.0535	0.044*	
C4	−0.0923 (3)	0.4925 (3)	0.03236 (16)	0.0299 (8)	
H4	−0.1162	0.5174	−0.0091	0.036*	
C5	0.0310 (3)	0.4960 (2)	0.05629 (16)	0.0243 (8)	
H5	0.0917	0.5238	0.0309	0.029*	
C6	0.0675 (3)	0.4598 (2)	0.11655 (15)	0.0203 (7)	
C7	0.1985 (3)	0.4645 (2)	0.14193 (15)	0.0211 (7)	
C8	0.4045 (3)	0.3970 (3)	0.26468 (16)	0.0257 (8)	
H8	0.3563	0.3500	0.2863	0.031*	
C9	0.5224 (3)	0.4264 (3)	0.29441 (16)	0.0272 (8)	
C10	0.6075 (3)	0.4826 (2)	0.26093 (16)	0.0244 (7)	
C11	0.7181 (3)	0.5184 (3)	0.29505 (18)	0.0309 (8)	
C12	0.7422 (4)	0.4979 (3)	0.35821 (19)	0.0405 (10)	
H12	0.8168	0.5219	0.3804	0.049*	
C13	0.6570 (4)	0.4416 (3)	0.3905 (2)	0.0439 (11)	
H13	0.6743	0.4275	0.4346	0.053*	
C14	0.5498 (3)	0.4068 (3)	0.35949 (17)	0.0360 (9)	
H14	0.4928	0.3688	0.3821	0.043*	
C15	0.9083 (4)	0.6080 (4)	0.2875 (2)	0.0614 (14)	
H15A	0.8939	0.6510	0.3241	0.074*	
H15B	0.9524	0.6467	0.2566	0.074*	
H15C	0.9587	0.5494	0.3020	0.074*	
C16	0.5344 (3)	0.3336 (3)	0.11885 (17)	0.0279 (8)	
H16	0.5618	0.3288	0.1628	0.033*	
C17	0.5655 (3)	0.2574 (3)	0.07847 (18)	0.0323 (9)	
H17	0.6123	0.2008	0.0946	0.039*	
C18	0.5280 (4)	0.2640 (3)	0.01462 (19)	0.0400 (10)	
H18	0.5497	0.2130	−0.0142	0.048*	
C19	0.4576 (3)	0.3469 (3)	−0.00668 (17)	0.0356 (9)	
H19	0.4300	0.3539	−0.0505	0.043*	
C20	0.4281 (3)	0.4198 (3)	0.03744 (16)	0.0290 (9)	
H20	0.3780	0.4757	0.0231	0.035*	
C21	0.4288 (3)	0.6714 (3)	0.05572 (16)	0.0272 (8)	
H21	0.3416	0.6592	0.0539	0.033*	
C22	0.4729 (4)	0.7378 (3)	0.01264 (17)	0.0346 (9)	
H22	0.4175	0.7704	−0.0182	0.042*	
C23	0.6008 (4)	0.7560 (3)	0.01530 (18)	0.0342 (9)	
H23	0.6348	0.8008	−0.0140	0.041*	
C24	0.6760 (4)	0.7082 (3)	0.06089 (18)	0.0346 (9)	
H24	0.7633	0.7204	0.0640	0.041*	
C25	0.6260 (3)	0.6423 (3)	0.10249 (17)	0.0280 (8)	
H25	0.6799	0.6091	0.1338	0.034*	
C26	0.2924 (3)	0.6392 (3)	0.24550 (17)	0.0311 (9)	
H26	0.2249	0.5971	0.2303	0.037*	
C27	0.2755 (4)	0.7074 (3)	0.29368 (19)	0.0407 (10)	
H27	0.1967	0.7133	0.3106	0.049*	
C28	0.3734 (4)	0.7665 (3)	0.31680 (18)	0.0434 (11)	
H28	0.3639	0.8119	0.3510	0.052*	
C29	0.4846 (4)	0.7596 (3)	0.29042 (17)	0.0379 (9)	
H29	0.5528	0.8012	0.3053	0.045*	
C30	0.4966 (3)	0.6918 (2)	0.24201 (16)	0.0293 (8)	
H30	0.5742	0.6876	0.2236	0.035*	
N1	0.2366 (2)	0.4037 (2)	0.18851 (12)	0.0218 (6)	
N2	0.3587 (2)	0.4305 (2)	0.20981 (13)	0.0221 (7)	
N3	0.4673 (2)	0.4141 (2)	0.09866 (13)	0.0236 (7)	
N4	0.5022 (2)	0.6234 (2)	0.09999 (13)	0.0226 (7)	
N5	0.4026 (2)	0.6309 (2)	0.21957 (12)	0.0231 (7)	
O1	0.2693 (2)	0.53324 (17)	0.11795 (11)	0.0231 (6)	
O2	0.5914 (2)	0.50462 (18)	0.19950 (11)	0.0249 (6)	
O3	0.7924 (2)	0.5744 (2)	0.25842 (12)	0.0397 (7)	
O4	0.0449 (2)	0.4311 (2)	0.40770 (12)	0.0444 (7)	
O5	0.1830 (3)	0.4614 (2)	0.33036 (12)	0.0529 (8)	
O6	0.1873 (4)	0.5628 (3)	0.41864 (19)	0.1058 (16)	
O7	0.2518 (3)	0.3989 (4)	0.4303 (2)	0.1226 (19)	

### Atomic displacement parameters (Å^2^)

**Table:**

	*U*^11^	*U*^22^	*U*^33^	*U*^12^	*U*^13^	*U*^23^
Cl1	0.0324 (5)	0.0473 (6)	0.0278 (5)	−0.0055 (5)	0.0017 (4)	−0.0020 (4)
Co1	0.0196 (2)	0.0223 (2)	0.0201 (2)	0.0008 (2)	0.00069 (16)	0.0003 (2)
C1	0.031 (2)	0.039 (2)	0.029 (2)	0.0017 (17)	0.0005 (17)	0.0075 (18)
C2	0.0226 (19)	0.048 (2)	0.042 (2)	−0.0104 (19)	0.0074 (18)	0.001 (2)
C3	0.0205 (18)	0.036 (2)	0.053 (3)	−0.0018 (17)	−0.0017 (18)	−0.0009 (19)
C4	0.0265 (19)	0.035 (2)	0.027 (2)	0.0033 (17)	−0.0041 (16)	0.0033 (16)
C5	0.0213 (18)	0.0247 (19)	0.0279 (19)	−0.0019 (15)	0.0080 (15)	0.0005 (15)
C6	0.0212 (16)	0.0208 (18)	0.0190 (17)	0.0007 (14)	0.0019 (13)	−0.0030 (14)
C7	0.0209 (17)	0.0215 (18)	0.0212 (18)	−0.0024 (14)	0.0044 (14)	−0.0049 (15)
C8	0.0300 (19)	0.025 (2)	0.023 (2)	0.0059 (16)	0.0062 (16)	0.0068 (16)
C9	0.0233 (18)	0.030 (2)	0.028 (2)	0.0036 (16)	−0.0002 (15)	0.0020 (16)
C10	0.0230 (18)	0.0251 (18)	0.0240 (19)	0.0065 (16)	−0.0047 (14)	0.0001 (16)
C11	0.0258 (19)	0.030 (2)	0.036 (2)	0.0004 (16)	−0.0044 (16)	0.0023 (17)
C12	0.036 (2)	0.049 (3)	0.034 (2)	0.0016 (19)	−0.0123 (17)	0.0012 (19)
C13	0.040 (2)	0.062 (3)	0.028 (2)	−0.001 (2)	−0.0042 (18)	0.003 (2)
C14	0.033 (2)	0.044 (2)	0.030 (2)	0.0042 (18)	−0.0036 (17)	0.0096 (18)
C15	0.042 (3)	0.070 (3)	0.068 (3)	−0.025 (2)	−0.017 (2)	0.019 (3)
C16	0.0209 (17)	0.029 (2)	0.034 (2)	0.0002 (16)	0.0039 (15)	0.0010 (16)
C17	0.028 (2)	0.024 (2)	0.045 (2)	0.0037 (16)	0.0055 (18)	−0.0034 (17)
C18	0.041 (2)	0.032 (2)	0.048 (3)	0.0020 (19)	0.015 (2)	−0.0157 (19)
C19	0.043 (2)	0.039 (2)	0.025 (2)	−0.0064 (19)	0.0038 (17)	−0.0085 (17)
C20	0.034 (2)	0.0222 (19)	0.030 (2)	−0.0026 (17)	0.0018 (17)	−0.0033 (16)
C21	0.0302 (19)	0.024 (2)	0.027 (2)	−0.0002 (16)	0.0018 (16)	0.0007 (16)
C22	0.044 (2)	0.035 (2)	0.024 (2)	0.0009 (19)	−0.0030 (18)	0.0046 (17)
C23	0.046 (2)	0.027 (2)	0.031 (2)	−0.006 (2)	0.0118 (19)	0.0030 (18)
C24	0.032 (2)	0.033 (2)	0.039 (2)	−0.0063 (18)	0.0079 (17)	−0.0001 (18)
C25	0.0244 (19)	0.030 (2)	0.030 (2)	−0.0004 (16)	0.0044 (16)	0.0021 (16)
C26	0.035 (2)	0.030 (2)	0.029 (2)	0.0049 (17)	0.0048 (17)	−0.0030 (16)
C27	0.051 (3)	0.032 (2)	0.041 (2)	0.005 (2)	0.021 (2)	−0.0032 (19)
C28	0.071 (3)	0.033 (3)	0.027 (2)	0.000 (2)	0.009 (2)	−0.0062 (18)
C29	0.049 (2)	0.030 (2)	0.033 (2)	−0.0051 (19)	−0.0034 (19)	−0.0027 (17)
C30	0.034 (2)	0.0231 (19)	0.030 (2)	−0.0036 (17)	0.0001 (16)	−0.0008 (16)
N1	0.0174 (14)	0.0254 (16)	0.0220 (15)	−0.0027 (12)	−0.0023 (11)	0.0008 (12)
N2	0.0221 (15)	0.0241 (17)	0.0204 (16)	0.0015 (13)	0.0032 (12)	−0.0018 (13)
N3	0.0206 (15)	0.0267 (17)	0.0237 (17)	0.0005 (13)	0.0039 (13)	0.0009 (13)
N4	0.0235 (15)	0.0204 (16)	0.0240 (16)	−0.0005 (13)	0.0023 (13)	−0.0032 (12)
N5	0.0279 (16)	0.0214 (16)	0.0201 (16)	0.0015 (14)	0.0022 (13)	0.0005 (13)
O1	0.0204 (13)	0.0250 (14)	0.0240 (14)	−0.0020 (10)	0.0016 (11)	0.0028 (11)
O2	0.0183 (12)	0.0336 (15)	0.0223 (14)	−0.0002 (10)	−0.0011 (11)	0.0032 (11)
O3	0.0259 (13)	0.0492 (18)	0.0421 (16)	−0.0111 (13)	−0.0093 (12)	0.0085 (14)
O4	0.0353 (15)	0.0655 (19)	0.0331 (15)	−0.0087 (14)	0.0075 (12)	−0.0004 (13)
O5	0.0571 (18)	0.073 (2)	0.0310 (16)	−0.0146 (16)	0.0199 (14)	−0.0125 (14)
O6	0.170 (4)	0.074 (3)	0.082 (3)	−0.071 (3)	0.058 (3)	−0.050 (2)
O7	0.052 (2)	0.178 (5)	0.137 (4)	0.029 (3)	0.003 (2)	0.094 (4)

### Geometric parameters (Å, °)

**Table:**

Cl1—O7	1.392 (4)	C15—O3	1.416 (4)
Cl1—O5	1.413 (3)	C15—H15A	0.9800
Cl1—O6	1.412 (4)	C15—H15B	0.9800
Cl1—O4	1.427 (2)	C15—H15C	0.9800
Co1—O2	1.866 (2)	C16—N3	1.338 (4)
Co1—N2	1.864 (3)	C16—C17	1.377 (5)
Co1—O1	1.898 (2)	C16—H16	0.9500
Co1—N5	1.957 (3)	C17—C18	1.375 (5)
Co1—N3	1.977 (3)	C17—H17	0.9500
Co1—N4	1.987 (3)	C18—C19	1.387 (5)
C1—C2	1.381 (5)	C18—H18	0.9500
C1—C6	1.391 (4)	C19—C20	1.393 (5)
C1—H1A	0.9500	C19—H19	0.9500
C2—C3	1.370 (5)	C20—N3	1.326 (4)
C2—H2	0.9500	C20—H20	0.9500
C3—C4	1.388 (5)	C21—N4	1.334 (4)
C3—H3	0.9500	C21—C22	1.374 (5)
C4—C5	1.381 (5)	C21—H21	0.9500
C4—H4	0.9500	C22—C23	1.394 (5)
C5—C6	1.384 (5)	C22—H22	0.9500
C5—H5	0.9500	C23—C24	1.361 (5)
C6—C7	1.468 (4)	C23—H23	0.9500
C7—O1	1.314 (4)	C24—C25	1.376 (5)
C7—N1	1.309 (4)	C24—H24	0.9500
C8—N2	1.297 (4)	C25—N4	1.352 (4)
C8—C9	1.423 (5)	C25—H25	0.9500
C8—H8	0.9500	C26—N5	1.349 (4)
C9—C14	1.402 (5)	C26—C27	1.381 (5)
C9—C10	1.411 (4)	C26—H26	0.9500
C10—O2	1.324 (4)	C27—C28	1.369 (5)
C10—C11	1.422 (4)	C27—H27	0.9500
C11—O3	1.372 (4)	C28—C29	1.362 (5)
C11—C12	1.361 (5)	C28—H28	0.9500
C12—C13	1.400 (5)	C29—C30	1.372 (5)
C12—H12	0.9500	C29—H29	0.9500
C13—C14	1.360 (5)	C30—N5	1.349 (4)
C13—H13	0.9500	C30—H30	0.9500
C14—H14	0.9500	N1—N2	1.399 (3)
			
O7—Cl1—O5	112.1 (2)	H15A—C15—H15B	109.5
O7—Cl1—O6	109.1 (3)	O3—C15—H15C	109.5
O5—Cl1—O6	108.3 (2)	H15A—C15—H15C	109.5
O7—Cl1—O4	107.9 (2)	H15B—C15—H15C	109.5
O5—Cl1—O4	109.92 (16)	N3—C16—C17	122.6 (3)
O6—Cl1—O4	109.6 (2)	N3—C16—H16	118.7
O2—Co1—N2	93.38 (11)	C17—C16—H16	118.7
O2—Co1—O1	175.67 (11)	C18—C17—C16	119.4 (4)
N2—Co1—O1	82.99 (11)	C18—C17—H17	120.3
O2—Co1—N5	89.42 (11)	C16—C17—H17	120.3
N2—Co1—N5	89.80 (12)	C17—C18—C19	118.4 (3)
O1—Co1—N5	92.92 (11)	C17—C18—H18	120.8
O2—Co1—N3	89.06 (11)	C19—C18—H18	120.8
N2—Co1—N3	89.56 (11)	C18—C19—C20	118.7 (3)
O1—Co1—N3	88.55 (10)	C18—C19—H19	120.7
N5—Co1—N3	178.31 (13)	C20—C19—H19	120.7
O2—Co1—N4	90.21 (11)	N3—C20—C19	122.5 (3)
N2—Co1—N4	176.30 (13)	N3—C20—H20	118.7
O1—Co1—N4	93.39 (11)	C19—C20—H20	118.7
N5—Co1—N4	91.12 (10)	N4—C21—C22	123.3 (3)
N3—Co1—N4	89.61 (12)	N4—C21—H21	118.3
C2—C1—C6	120.3 (3)	C22—C21—H21	118.3
C2—C1—H1A	119.9	C21—C22—C23	118.5 (4)
C6—C1—H1A	119.9	C21—C22—H22	120.8
C3—C2—C1	120.4 (3)	C23—C22—H22	120.8
C3—C2—H2	119.8	C24—C23—C22	118.5 (4)
C1—C2—H2	119.8	C24—C23—H23	120.8
C2—C3—C4	120.2 (3)	C22—C23—H23	120.8
C2—C3—H3	119.9	C23—C24—C25	120.2 (3)
C4—C3—H3	119.9	C23—C24—H24	119.9
C5—C4—C3	119.3 (3)	C25—C24—H24	119.9
C5—C4—H4	120.3	N4—C25—C24	121.8 (3)
C3—C4—H4	120.3	N4—C25—H25	119.1
C4—C5—C6	121.1 (3)	C24—C25—H25	119.1
C4—C5—H5	119.4	N5—C26—C27	121.4 (4)
C6—C5—H5	119.4	N5—C26—H26	119.3
C5—C6—C1	118.7 (3)	C27—C26—H26	119.3
C5—C6—C7	120.9 (3)	C28—C27—C26	119.5 (4)
C1—C6—C7	120.4 (3)	C28—C27—H27	120.3
O1—C7—N1	123.9 (3)	C26—C27—H27	120.3
O1—C7—C6	117.3 (3)	C29—C28—C27	119.5 (4)
N1—C7—C6	118.7 (3)	C29—C28—H28	120.2
N2—C8—C9	124.0 (3)	C27—C28—H28	120.2
N2—C8—H8	118.0	C28—C29—C30	119.0 (4)
C9—C8—H8	118.0	C28—C29—H29	120.5
C14—C9—C10	119.5 (3)	C30—C29—H29	120.5
C14—C9—C8	119.3 (3)	N5—C30—C29	122.6 (3)
C10—C9—C8	121.0 (3)	N5—C30—H30	118.7
O2—C10—C9	124.4 (3)	C29—C30—H30	118.7
O2—C10—C11	117.3 (3)	C7—N1—N2	108.2 (3)
C9—C10—C11	118.3 (3)	C8—N2—N1	118.6 (3)
O3—C11—C12	125.7 (3)	C8—N2—Co1	126.4 (3)
O3—C11—C10	113.5 (3)	N1—N2—Co1	114.7 (2)
C12—C11—C10	120.9 (3)	C20—N3—C16	118.4 (3)
C11—C12—C13	120.0 (4)	C20—N3—Co1	121.1 (2)
C11—C12—H12	120.0	C16—N3—Co1	120.5 (2)
C13—C12—H12	120.0	C21—N4—C25	117.7 (3)
C14—C13—C12	120.7 (4)	C21—N4—Co1	121.2 (2)
C14—C13—H13	119.7	C25—N4—Co1	121.0 (2)
C12—C13—H13	119.7	C30—N5—C26	118.0 (3)
C13—C14—C9	120.7 (4)	C30—N5—Co1	120.1 (2)
C13—C14—H14	119.7	C26—N5—Co1	121.5 (2)
C9—C14—H14	119.7	C7—O1—Co1	109.2 (2)
O3—C15—H15A	109.5	C10—O2—Co1	121.7 (2)
O3—C15—H15B	109.5	C11—O3—C15	117.3 (3)
